# Metformin-Associated Lactic Acidosis (MALA): When a Common Drug Turns Catastrophic

**DOI:** 10.7759/cureus.89292

**Published:** 2025-08-03

**Authors:** Lauren E Kessler, Dheerja Sachdeva, Rohan Singhal, Kevin O Wortman II, Rekha Lall

**Affiliations:** 1 Nephrology, Edward Via College of Osteopathic Medicine, Auburn, USA; 2 Internal Medicine, Hamdard Institute of Medical Sciences and Research, New Delhi, IND; 3 Internal Medicine, Atal Bihari Vajpayee Institute of Medical Sciences and Dr. Ram Manohar Lohia Hospital, New Delhi, IND; 4 Nephrology, Tulane University School of Medicine, New Orleans, USA; 5 Internal Medicine, Edward Via College of Osteopathic Medicine, Auburn, USA

**Keywords:** acute kidney injury, case report, hemodialysis, metformin, metformin-associated lactic acidosis, metformin toxicity, type 2 diabetes mellitus

## Abstract

Metformin, a first-line agent in the treatment of type 2 diabetes mellitus, is widely favored for its efficacy and safety profile; however, under conditions of impaired renal function, it can lead to metformin-associated lactic acidosis (MALA), a rare but life-threatening complication. The diagnosis requires careful exclusion of other causes of lactic acidosis, such as sepsis or hypoperfusion, which can mimic MALA. We present a case of an 88-year-old female with type 2 diabetes and chronic kidney disease (CKD) stage III who developed severe lactic acidosis and encephalopathy in the setting of acute kidney injury and recent infection. The patient's condition rapidly deteriorated despite broad-spectrum antibiotics and hemodynamic support. Emergent dialysis resulted in significant clinical and biochemical improvement, supporting a diagnosis of MALA. This case emphasizes the importance of early recognition of MALA, particularly in vulnerable populations with evolving renal dysfunction. We present the pathophysiology, diagnostic approach, and management strategies for MALA. The timely initiation of renal replacement therapy remains critical for reversing metabolic derangements and improving outcomes.

## Introduction

Metformin, a biguanide, is a first-line treatment for type 2 diabetes due to its safety, efficacy, and widespread availability. It has been shown to reduce cardiovascular events, microvascular complications, and overall mortality in patients with diabetes [1]. Metformin works by decreasing hepatic glucose production (gluconeogenesis) without increasing insulin levels, making hypoglycemia a rare side effect [2].

Historically, *Galega officinalis*, a plant rich in guanidine, was used in the Middle Ages to treat diabetes. In 1918, guanidine was found to lower blood glucose but was too toxic for therapeutic use. The development of biguanides in 1929 provided similar glucose-lowering effects with a better safety profile. Metformin was first studied in the 1950s and received FDA approval in 1995. By 2012, it became the recommended first-line therapy for non-insulin-dependent diabetes and is now used by over 150 million people annually [3].

Although metformin is generally well tolerated, common side effects include gastrointestinal symptoms, such as bloating, discomfort, and diarrhea. A rare but serious complication is metformin-associated lactic acidosis (MALA), defined as lactic acidosis (arterial pH <7.35 and lactate >5 mmol/L) occurring in patients taking metformin [1].

Phenformin, another biguanide, was withdrawn from the U.S. market in 1997 due to a significantly higher risk of lactic acidosis, with an incidence of up to two cases per 1,000 users annually [4]. Phenformin posed a 10- to 20-fold higher risk than metformin, due to its stronger inhibition of mitochondrial function and lactate metabolism [5]. Additionally, nearly 10% of Caucasians have a genetic variant impairing phenformin metabolism, further increasing toxicity risk [6].

While metformin toxicity is rare, it remains a concern, particularly in patients with impaired kidney function or those who overdose [1]. Metformin increases lactate levels by enhancing glucose conversion to lactate in the small intestine and by reducing hepatic clearance of lactate through suppression of gluconeogenesis [2,7]. Since metformin is excreted via the kidneys, renal function must be monitored closely to prevent drug accumulation [8]. Current guidelines recommend using estimated glomerular filtration rate (eGFR) rather than serum creatinine to guide metformin use. Discontinuation is advised at eGFR <30 mL/min/1.73 m², with dose reduction and closer monitoring recommended at eGFR <45 mL/min/1.73 m² [1,2].

Clinical features of MALA include gastrointestinal symptoms (e.g., abdominal pain, vomiting, diarrhea), respiratory distress, tachycardia, hypotension, and altered mental status. Severe cases may progress to ventricular arrhythmias, respiratory failure, multiorgan dysfunction, and vasoplegic shock [9]. Mortality rates from MALA are reported between 20% and 36% [10]. Diagnosis is primarily clinical, as serum metformin levels are not always timely or reliable indicators of severity [11].

Management is largely supportive. Nephrology consultation should be obtained promptly, and hemodialysis should be considered for patients with arterial pH ≤7.0, serum lactate >20 mmol/L, or those who fail to improve with conservative treatment [12]. In severe acidemia (pH ≤7.2), especially when accompanied by acute kidney injury, intravenous sodium bicarbonate may be used to maintain physiologic pH. In cases of acute overdose, activated charcoal may be considered if the patient presents early and the airway is protected [13].

## Case presentation

An 88-year-old female with a history of well-controlled type 2 diabetes (most recent HbA1c 6.3%) with peripheral neuropathy and stage III chronic kidney disease, as well as lumbar degenerative disc disease with a compression fracture, presented to the emergency department (ED), encephalopathic and lethargic. She was discharged from the hospital one week prior for a non-displaced left ankle fracture and urinary tract infection (UTI) that appeared to have resolved with IV ceftriaxone. She was discharged to an acute rehab and did well for the first three to four days. Her home medications included oxycodone 10 mg every 8 h and fentanyl 12 μg per h transdermal film every 72 h for pain, losartan, rosuvastatin, pregabalin, and metformin 500 mg twice per day. Her caregivers noted that she had decreased oral intake while at the facility. On the day of presentation, she appeared encephalopathic and dyspneic and was brought to the ED via emergency medical services.

In the ED, she was afebrile with an oral temperature of 36.7°C, tachycardic, tachypneic, and hypotensive (mean arterial pressure 50s mmHg) with a baseline blood pressure of 160/93 mmHg, requiring 2 L nasal cannula, and a body mass index (BMI) of 49.54 kg/m², and glucose was 75 mg/dL. On examination, she was lethargic and in mild pulmonary distress. She was alert to person and place but not oriented to time. Heart sounds were regular in rate and rhythm. Lungs were clear to auscultation bilaterally. The abdomen was obese but soft, non-tender, non-distended, with normoactive bowel sounds. There were no noted focal neurologic deficits. Her mental processing appeared delayed. There was no jugular venous distention or peripheral edema. Pertinent labs are included below (Table 1).

**Table 1:** Laboratory values at the time of admission. FEU: fibrinogen equivalent units; PaCO₂: partial pressure of CO₂; PaO₂: partial pressure of O₂; HCO₃⁻: bicarbonate; FiO₂: fraction of inspired oxygen

Laboratory test	Patient value	Reference range	Units
Serum potassium	6.5	3.5-5.0	mEq/L
Anion gap	17	8-16	mEq/L
Serum creatinine	4.8	0.6-1.2	mg/dL
Baseline serum creatinine	1.0	0.6-1.2	mg/dL
Estimated glomerular filtration rate (eGFR)	9	>60	mL/min/1.73 m²
Baseline eGFR	55	>60	mL/min/1.73 m²
High-sensitivity troponin	33	<14	ng/L
Lactic acid	6.0	0.5-2.2	mmol/L
White blood cell count (WBC)	17.7	4.0-11.0	×10³/μL
D-dimer	653	<500	ng/mL FEU
Arterial blood gas (ABG)			
pH	6.98	7.35-7.45	-
PaCO₂	46	35-45	mmHg
PaO₂	84	80-100	mmHg
HCO₃⁻	10	22-28	mEq/L
FiO₂	32	Room air = 21	%

The electrocardiogram showed sinus tachycardia. Echocardiogram revealed a normal-sized left ventricle with mild concentric left ventricular hypertrophy and preserved systolic function, with an ejection fraction of 55-65%. No wall motion abnormalities were observed. Moderate aortic stenosis, a known preexisting condition, was noted. Respiratory BioFire panel (Salt Lake City, UT: BioFire Diagnostics) was negative for viral pathogens. Urinalysis results are included in Table 2.

**Table 2:** Urinalysis values at the time of admission.

Urinalysis parameter	Patient value	Reference range/interpretation
Leukocyte esterase	Moderate	Negative
Nitrite	Negative	Negative
White blood cells (WBCs)	10 per high-power field	0-5 per high-power field
Bacteria	Trace	None
Yeast	Moderate	None
Ketones	Negative	Negative

**Figure 1:**
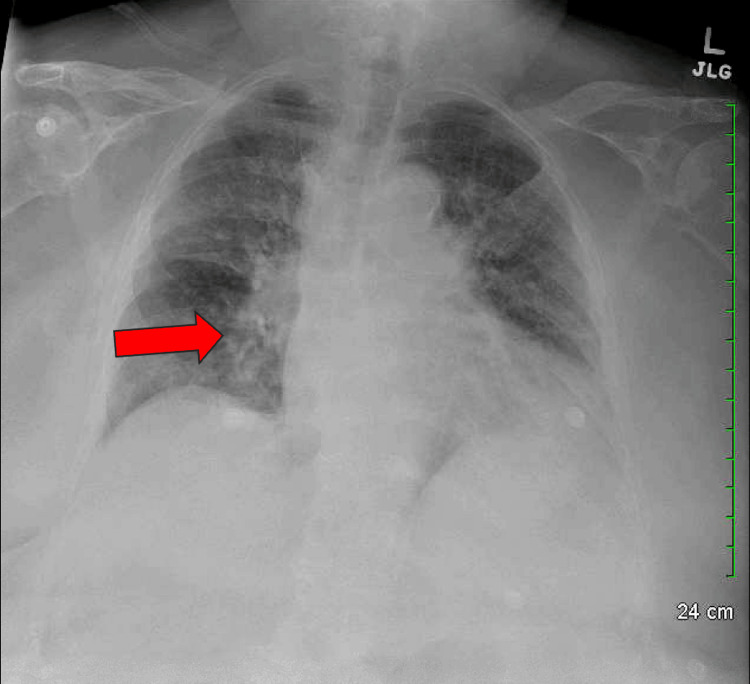
Chest X-ray on admission. The arrow indicates cardiomegaly and bilateral perihilar opacities, consistent with pulmonary edema.

**Figure 2:**
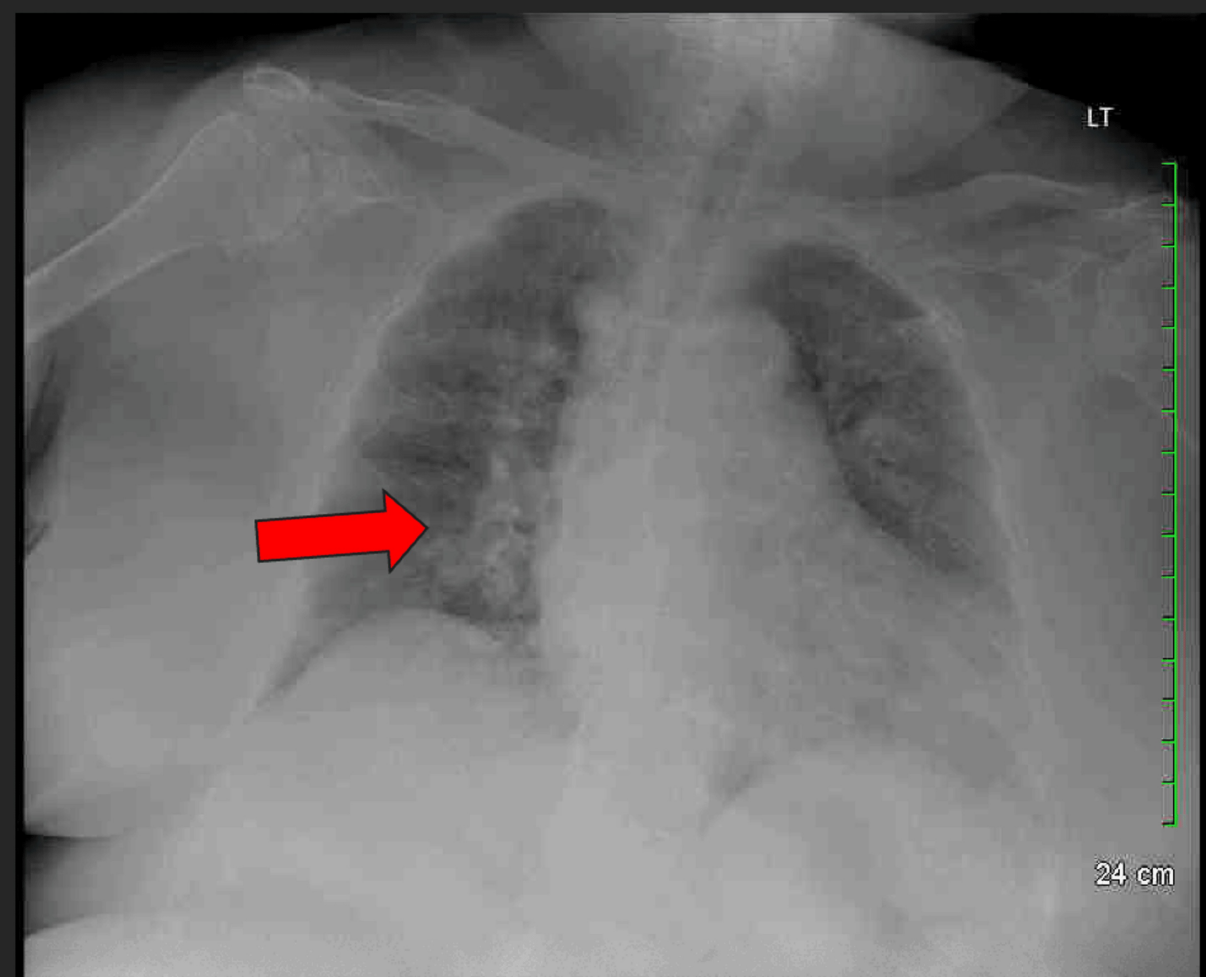
Chest X-ray from two weeks prior. The arrow indicates the location and appearance of the opacities with no significant change, suggesting a chronic or recurrent process.

Figure 1 shows cardiomegaly and bilateral perihilar opacities suggestive of pulmonary edema, unchanged in Figure 2, taken two weeks earlier, indicating a chronic or recurrent process.

Given her recent hospitalization, abnormal urinalysis, new chest imaging findings, leukocytosis, hypotension, and elevated lactate, she was initially treated for presumed septic shock secondary to hospital-acquired pneumonia or recurrent UTI. Broad-spectrum antibiotics (vancomycin, piperacillin-tazobactam, and azithromycin) and corticosteroids were initiated.

Cultures of blood, urine, and sputum were obtained, and an abdominal ultrasound showed no evidence of urinary obstruction. However, the progression of her lactic acidosis to 10.0 mmol/L despite aggressive fluid resuscitation, along with severe acidemia and acute kidney injury, raised concern for metformin-associated lactic acidosis (MALA), particularly in the context of known metformin use, poor oral intake, no source of infection, and negative cultures. Nephrology was consulted, and emergent hemodialysis was initiated. Despite supportive care and vasopressor therapy with norepinephrine, her lactic acid remained elevated until after dialysis, which resulted in a downtrend to 2.5 mmol/L and subsequently 1.3 mmol/L within 6 h. Follow-up imaging, including CT of the brain, chest, and abdomen/pelvis, showed no acute intracranial pathology, no evidence of pulmonary embolism, and no abdominal or mesenteric ischemia. Fractional excretion of sodium was 1.2%, and fractional excretion of urea was 8.6%, consistent with prerenal azotemia. Her high-sensitivity troponin continued to rise, peaking at 7,437 ng/L. Echocardiogram revealed no wall motion abnormalities, and cardiology attributed the rise in troponin to a type 2 non-ST elevation myocardial infarction secondary to hypoperfusion. She underwent two additional dialysis sessions with gradual improvement in mentation, and she was alert and oriented to person, place, and time, though she remained anuric, and her respiratory status remained unchanged. After discussion with the patient and her family, the decision was made to forgo outpatient dialysis. She was transitioned to home hospice and passed away eight days later.

## Discussion

Metformin toxicity, predominantly manifesting as metformin-associated lactic acidosis (MALA), represents a critical clinical condition with significant morbidity and mortality. Our case aligns with extensive evidence indicating that most toxicity arises from chronic therapeutic use complicated by acute kidney injury or from acute overdose.

A large meta-summary of 242 cases found that 76.4% of patients developed toxicity during chronic metformin use, often triggered by renal impairment or other precipitating factors, such as dehydration or sepsis, while 23.6% resulted from acute overdose, frequently suicidal in intent. The median acute ingested dose was approximately 42.5 g, with a mortality rate near 19.3% in overdose cases. This highlights that both chronic accumulation and acute ingestion can produce life-threatening lactic acidosis [10]. Several case reports illustrate the spectrum of metformin toxicity. One patient with type 2 diabetes on metformin developed severe lactic acidosis and multiorgan failure triggered by acute kidney injury caused by pancreatitis and sepsis, ultimately resulting in fatal MALA despite aggressive treatment, highlighting how acute illnesses can precipitate toxicity in chronic metformin users [14]. In contrast, another patient who developed MALA following prolonged dehydration and acute kidney injury showed rapid improvement after timely initiation of hemodialysis, underscoring the vital role of early recognition and prompt renal replacement therapy in the effective management of metformin toxicity [15].

Acute overdose cases often involve intentional ingestion with severe outcomes. For example, an adolescent who ingested 8 g developed fatal multiorgan failure despite hemodialysis and supportive care, illustrating the high mortality risk in such cases [16]. In contrast, another patient who overdosed on more than 50 g developed severe metabolic acidosis, hyperlactatemia, hypotension, and altered consciousness. Although initial treatment was insufficient, prolonged hemodialysis combined with continuous hemofiltration successfully reversed the acidosis and stabilized the patient [17]. This emphasizes that even severe metabolic derangements can be effectively managed with early recognition and aggressive renal replacement therapy, underscoring the critical role of prompt dialysis in improving outcomes in severe metformin toxicity. Another case of a non-diabetic girl overdosing on a large dose of metformin presented with severe lactic acidosis and a rapidly rising, extreme hyperglycemia (peaking at 497 mg/dL), likely due to acute pancreatitis thereby highlighting a rare finding in metformin toxicity, especially in non-diabetic patients and highlighting that pancreatitis-induced insulin deficiency can complicate the clinical course and signal severe toxicity [18].

Management centers on supportive care and rapid removal of metformin and lactate via renal replacement therapy (RRT). Almost 70% of patients required RRT, predominantly hemodialysis, which has been shown to improve survival by correcting acidosis and removing metformin. Vasopressors and mechanical ventilation were also frequently needed. Sodium bicarbonate was used in 2/3 of cases, although its benefit remains debated [10]. Despite treatment advances, mortality remains substantial, around 20%, particularly in patients presenting with shock, multiorgan failure, or profound acidosis. This underscores the importance of early recognition, close monitoring of renal function in metformin users, and patient education to avoid precipitating factors [19].

## Conclusions

Metformin toxicity presents a diagnostic and therapeutic challenge, with outcomes heavily dependent on timely intervention. This case and the literature review emphasize that clinicians must maintain a high index of suspicion for MALA in patients on metformin who develop metabolic acidosis, especially in the context of renal dysfunction or overdose. Prompt supportive care and renal replacement therapy remain the cornerstones of management to reduce mortality.

Early recognition of subtle clinical changes, such as encephalopathy and hypotension in at-risk patients, is crucial for initiating timely intervention. Regular monitoring of renal function in elderly patients on metformin, particularly during acute illnesses, such as infection or dehydration, can help prevent catastrophic outcomes. Preventative measures, such as patient education on dehydration risks and regular eGFR monitoring, are critical to reducing MALA incidence in high-risk populations. This case highlights the importance of individualized medication review and interprofessional collaboration, including early nephrology involvement, to improve outcomes in vulnerable populations.
